# Twentieth Century Practice

**Published:** 1898-04

**Authors:** 


TWENTIETH CENTURY PRACTICE. An International Encyclopedia of Modern Medical Science. By leading authorities of Europe and America. Edited by Thomas L. Stedman, M. D., New York City. In twenty volumes. Volume XIII, "Infectious Diseases." New York: William Wood & Company. 1898.

Dr. Victor C. Vaughan, of Ann Arbor, contributes the first article in this volume; the subjects, viz: ptomaines, toxins and leucomaines, are very important ones. In introducing the subject of bacterial poisons the author uses the following very forcible, and we think very truthful, language: "That the infectious diseases are caused by certain micro-organisms has been positively demonstrated" * * * "It is true that in these last years of the nineteenth century there are a few who still question the causal relationship of germs to disease, but professionally these live in a past now so remote that we may consider them as only of archeological interest." Besides the bacterial poisons Dr. Vaughan discusses ptomaines; food, fish, meat, milk, cheese, vegetable and other poisons. The entire article covers 130 pages and is a most valuable contribution to the literature of the subjects discussed.

Infection and immunity is the subject of an unusually good article by Dr. Harold C. Ernst, of Boston. This chapter is a very lengthy one—140 pages—about 45 pages of it being devoted to the serum treatment.

The late Mr. Ernest Hart and Dr. Solomon C. Smith of London, have contributed an article on "Water-borne Diseases." The subject is divided into two parts: 1st, diseases caused by non-living matter—as lead, zinc, copper, arsenic, etc., and ptomain poisoning. 2nd, diseases caused by living organisms. This includes the entozoa, typhoid fever, cholera, malaria, dysentery, diarrhœa and yellow fever.

The duration of the periods of incubation and infectiousness in acute specific diseases, forms the subject of a 20-page article by Dr. Dawson Williams, of London.

Dr. John William Moore, of Dublin, contributes an interesting paper on small-pox, and Dr. P. Brouardel, of Paris, one on vaccina.

The last chapter in the volume is on the subject of mumps, and it was written by Dr. Jules Comby, of Paris.

The publishers of the Twentieth Century Practice are to be congratulated on being able to bring out the successive volumes of this superb work so near on the time first announced. The thirteen volumes already published show up well, and any physician who may be so fortunate as to own the set may justly feel proud of it. H.

